# Short-term efficacy and safety of a lower dose of polyethylene glycol recombinant human growth hormone in children with growth hormone deficiency: A randomized, dose-comparison study

**DOI:** 10.3389/fphar.2022.955809

**Published:** 2022-08-11

**Authors:** Zhouhong Jiang, Xuefeng Chen, Guanping Dong, Yin Lou, Jianping Zhang, Xinran Cheng, Jiayan Pan, Wei Liao, Jinzhun Wu, Xiaodong Huang, Xianjiang Jin, Deyun Liu, Ting Zeng, Shunye Zhu, Qin Dong, Xiaoming Luo, Dan Lan, Lizhi Cao, Xingxing Zhang, Jing Liu, Mingjuan Dai, Manyan Zhang, Li Liu, Junhua Dong, Dongmei Zhao, Shaoqing Ni, Junfen Fu

**Affiliations:** ^1^ Department of Pharmacy, The Children’s Hospital, Zhejiang University School of Medicine, National Clinical Research Center for Child Health, Hangzhou, China; ^2^ Department of Endocrinology, The Children’s Hospital, Zhejiang University School of Medicine, National Clinical Research Center for Child Health, Hangzhou, China; ^3^ Department of Pediatrics, Ningbo Women’s and Children’s Hospital, Ningbo, China; ^4^ Department of Pediatric Endocrine Genetics and Metabolism, Chengdu Women’s and Children’s Center Hospital, Chengdu, China; ^5^ Department of Pediatrics, Wuhu First People’s Hospital, Wuhu, China; ^6^ Department of Pediatrics, First Affiliated Hospital of Army Medical University (Third Military Medical University), Chongqing, China; ^7^ Department of Pediatrics, The First Affiliated Hospital of Xiamen University, Xiamen, China; ^8^ Department of Endocrinology and Genetics, Shanghai Children’s Medical Center, Shanghai Jiaotong University School of Medicine, Shanghai, China; ^9^ Department of Genetics and Endocrinology, The Second Affiliated Hospital and Yuying Children’s Hospital of Wenzhou Medical University, Wenzhou, China; ^10^ Department of Pediatrics, The Second Affiliated Hospital of Anhui Medical University, Hefei, China; ^11^ Department of Child Health Care, Liuzhou Maternity and Child Healthcare Hospital, Liuzhou, China; ^12^ Department of Pediatrics, The Third Affiliated Hospital of Sun Yat-Sen University, Guangzhou, China; ^13^ Department of Pediatrics, Zhejiang Provincial Hospital of Chinese Medicine, Hangzhou, China; ^14^ Department of Pediatrics, Zhejiang Provincial People’s Hospital, People’s Hospital of Hangzhou Medical College, Hangzhou, China; ^15^ Department of Pediatrics, The First Affiliated Hospital of Guangxi Medical University, Nanning, China; ^16^ Department of Pediatrics, Xiangya Hospital, Central South University, Changsha, China; ^17^ Department of Pediatrics, The Second Xiangya Hospital, Central South University, Changsha, China; ^18^ Department of Pediatrics, Changchun Children’s Hospital, Changchun, China; ^19^ Department of Pediatrics, Hangzhou First People’s Hospital, Hangzhou, China; ^20^ Department of Pediatrics, Shaoxing Second Hospital, Shaoxing, China; ^21^ Department of Genetics and Endocrinology, Guangzhou Women and Children’s Medical Center, Guangzhou, China; ^22^ Department of Pediatrics, Qilu Hospital of Shandong University, Jinan, China; ^23^ Pediatric Research Institute, Qilu Children’s Hospital of Shandong University, Jinan, China; ^24^ National Clinical Trial Institute, The Children’s Hospital, Zhejiang University School of Medicine, National Clinical Research Center for Child Health, Hangzhou, China; ^25^ Research Center for Clinical Pharmacy, Zhejiang University, Hangzhou, China; ^26^ The Children’s Hospital, Zhejiang University School of Medicine, National Clinical Research Center for Child Health, Hangzhou, China

**Keywords:** PEG-rhGH, GHD, IGF-1, dose, children

## Abstract

**Objective:** Polyethylene glycol recombinant human growth hormone (PEG-rhGH, Jintrolong^®^) is the first long-acting rhGH preparation that is approved to treat children with growth hormone deficiency (GHD) in China. Clinical experience with dose selections of PEG-rhGH is scarce. The present study compared the efficacy and safety of a lower dose to increase dosing regimens of PEG-rhGH treatment.

**Methods:** A multicenter, randomized, open-label, dose-comparison clinical study was conducted to compare the improvements in the height standard deviation score (Ht SDS), height velocity (HV), insulin-like growth factor-1 (IGF-1) SDS, and safety profiles of children with GHD who are treated with 0.2 mg/kg/week of PEG-rhGH dose or 0.14 mg/kg/week for 26 weeks.

**Results:** Ht SDS, HV, and IGF-1 SDS increased significantly after PEG-rhGH treatment in the two dose groups (*p* < 0.05). The improvements of Ht SDS, HV, and IGF-1 SDS were more significant in the high-dose group than in the low-dose group (*p* < 0.05). Ht SDS improvement in low-dose group was not non-inferiority to that in the high-dose group (*p* = 0.2987). The incidences of adverse events were comparable between the two groups.

**Conclusion:** The improvements of Ht SDS, HV, and IGF-1 SDS were more significant in the high-dose group than in the low-dose group (*p* < 0.05). PEG-rhGH at the dose of 0.14 mg/kg/week was effective and safe for children with GHD.

**Clinical Trial Registration:** clinicaltrials.gov, identifier NCT02908958.

## Introduction

Recombinant human growth hormone (rhGH) has been used to treat growth hormone deficiency (GHD) in children for over 30 years with the aim of promoting linear growth (Richmond and Rogol, 2016; Collett-Solberg et al., 2019). The efficacy and safety of rhGH therapy has been demonstrated in many clinical trials (Shih et al., 1994; Peterkova et al., 2007; Slattery et al., 2014; Swerdlow et al., 2015; Rhie et al., 2019; Pfäffle et al., 2020; Backeljauw et al., 2021; Coutant et al., 2021). However, daily rhGH injections lead to poor adherence and decreased effectiveness. A recent meta-analysis reported that up to 71% of patients with GHD and their families were non-adherent to the prescribed treatment (Graham et al., 2018). As a result, several long-acting formulations of rhGH have been developed to reduce the frequency of administrations (Saenger and Mejia-Corletto, 2016; Miller et al., 2020).

Jintrolong^®^ (GeneScience Pharmaceuticals, Changchun, China), a polyethylene glycol rhGH (PEG-rhGH), is the first commercial long-acting rhGH preparation approved in China. Compared to daily rhGH, PEG-rhGH has a longer *T*_max_ and *t*_*1/2*_ and slower plasma clearance, which allows for weekly injection (Hou et al., 2016). Clinical studies have demonstrated the non-inferior efficacy and safety of PEG-rhGH compared to daily rhGH at an equivalent dose for the treatment of GHD (Luo et al., 2017; Qiao et al., 2019; Sun et al., 2021; Wang et al., 2021; Du et al., 2022). Notably, in a Phase III trial, PEG-rhGH treatment at 0.2 mg/kg/week was associated with greater increases in most of the efficacy endpoints, including height velocity (HV), height (Ht) standard deviation score (SDS), and insulin-like growth factor-1 (IGF-1) SDS, compared to daily rhGH dosing of 0.25 mg/kg/week (Luo et al., 2017). IGF-I has an effect on cell proliferation, and its increased serum concentration might be associated with an increased risk of common cancers (Renehan et al., 2004; Pfäffle, 2015). The change in the area under the concentration curve of IGF-1 after 7 days of PEG-rhGH injection at a dose of 0.2 mg/kg was 1.3 folds larger than that of rhGH at 0.25 mg/kg/week in a Phase I clinical trial (*p* = 0.059) (Hou et al., 2016). These results suggest that PEG-rhGH can be administrated at a lower dose to achieve comparable efficacy and safety.

Clinical experience with dose selections of PEG-rhGH is scarce. A PEG-rhGH dose of 0.14 mg/kg/week is equivalent to a daily rhGH dose of 0.12 IU/kg/d, which is within the recommended dose range for children with GHD. In addition, results of an animal study reported that in rats, a single PEG-rhGH dose of 0.14 mg/kg/week showed the same expected linear growth as a daily rhGH dose of 0.25 mg/kg/week (Zhang et al., 2012). Taken together, the present study aimed to compare the efficacy and safety of PEG-rhGH treatment at a dose of 0.14 mg/kg/week to 0.2 mg/kg/week in children with GHD.

## Materials and methods

### Study design and participants

This study was a multicenter, randomized, open-label, parallel-group, dose-comparison clinical trial that took place at 22 medical centers in China for 26 weeks. The study protocol was reviewed and approved by the Ethics Committee of the Children’s Hospital, Zhejiang University School of Medicine and other participating centers. The parents or legal guardians of all participating children signed informed consents. The study was conducted in accordance with the principles of the Declaration of Helsinki and the International Conference on Harmonization Good Clinical Practice guidelines.

Eligible participants were prepubertal GHD patients (Tanner stage 1) aged at 3 years or older who had not received any GH treatment for 6 months. GHD was diagnosed using the following criteria: 1) height below -2SD or the third percentile of the normal growth curve for children of the same chronological age (CA) and sex in China (Hui et al., 2009); 2) HV ≤ 5 cm/year; 3) serum GH peak <10 μg/L in two different GH stimulation tests (stimulation with insulin, L-dopa, glucagon, arginine, or clonidine); and 4) bone age (BA) below 10 years for boys and 9 years for girls, with a minimum of a 1 year delay compared to the CA. Key exclusion criteria included renal or hepatic impairment; positive results for hepatitis B virus test, hypersensitivity of the study drug, serious cardiopulmonary, hematologic diseases, systemic infections or immunocompromising disorder, familial history of malignant tumor, diabetes, and other abnormal growth syndromes (i.e., Turners, constitutional delay of puberty, Laron Syndrome, growth hormone receptor deficiency). Those who had participated in other clinical trials within the 3 months prior to enrollment were also excluded.

### Randomisation and masking

Patients were randomly assigned in a 1:1 ratio to randomized blocks (6 people per block) using a computer-generated random sequence to receive a PEG-rhGH dose of 0.14 mg/kg/week or 0.2 mg/kg/week. The study medicines and participant numbers were assigned in the forms of block multiples to each center. Each participant was assigned a unique medicine number. The investigators and parents/guardians were not masked to treatment allocation.

### Procedures

PEG-rhGH was subcutaneously injected at a fixed time of the day by patients or their parents/guardians, who were able to administrate PEG-rhGH after training. The injection sites could be the lateral upper arm, lateral thigh, or the abdomen except the periumbilical area; the two injection sites were to be more than 2 cm apart. Each administration date and time was carefully recorded on diary cards. The treatments lasted for 26 weeks, and three follow-up visits were scheduled at week 4, 13, and 26 ( ± 5 days) after treatment initiation. At each visit, height and body weight were measured by designated personnel at each center. Blood samples were collected for blood routine tests, blood biochemistry, blood lip, blood glucose, thyroid function, serum IGF-1 concentration and anti-drug antibodies. Pituitary magnetic resonance imaging and electrocardiography were performed at each center. BA radiography was performed using the Tanner-Whitehouse three method at baseline and at week 26. Participants were not to use other medicines that may affect the efficacy of PEG-rhGH, such as gonadotropin-releasing hormone analogs, androgens, anabolic hormones, or other drugs that affect growth and development.

### Outcomes

The primary efficacy outcome was the Ht SDS at week 26 after PEG-rhGH treatment. Secondary outcomes included HV and IGF-1 SDS at week 26 after PEG-rhGH treatment. Ht SDS and IGF-1 SDS were defined as the SD scores at each visit, based on the same CA and sex. HV was calculated as the height change per year. Safety was assessed by monitoring the adverse events (AEs), clinical symptoms, and laboratory tests at each visit. AEs were recorded, irrespective of their causal relationship to the treatment.

### Statistical analysis

A sample size of at least 191 per group was needed to achieve a power of 90% with an α level of 0.025 for a non-inferiority margin of −20% in Ht SDS change (Sun et al., 2021). We assumed a dropout rate of 20% and to guarantee the robustness of the results, a total of 900 patients were planned to recruit.

Efficacy analysis was performed at week 26 in the modified intention-to-treat (mITT) and per-protocol populations. The mITT population included all randomized patients who received at least one injection of PEG-rhGH and completed at least one follow-up visit. The per-protocol population comprised of all randomized patients from the mITT population who completed all follow-up visits and had no major protocol deviations. Safety analysis was performed on the safety set (SS), which included all randomized patients who received at least one injection and safety record.

Continuous variables are presented as mean ± SD and categorical variables are presented as frequency and percentage. To assess the changes in PEG-rhGH treatment before and after with-in groups, continuous variables were statistically analyzed by paired *t*-test if they were normally distributed and homogeneous; otherwise, a Wilcoxon rank-sum test was used. Missing data were imputed using the last-observation-carried-forward method. Changes between the two groups were analyzed by covariance (ANCOVA) with baseline as the covariate, taking the center effect into consideration. A chi-squared (χ^2^) test was used to compare enumeration data and ratios. Results were considered significant at *p* < 0.05. The non-inferiority of 0.14–0.2 mg/kg/week would be accepted if the lower limit of the two-sided 95% CI for the difference between the two dose groups was greater than the non-inferiority margin. All statistical analyses were performed using SAS (version 9.4, SAS Institute Inc. Cary, NC).

## Results

### Patient characteristics

**FIGURE 1:**
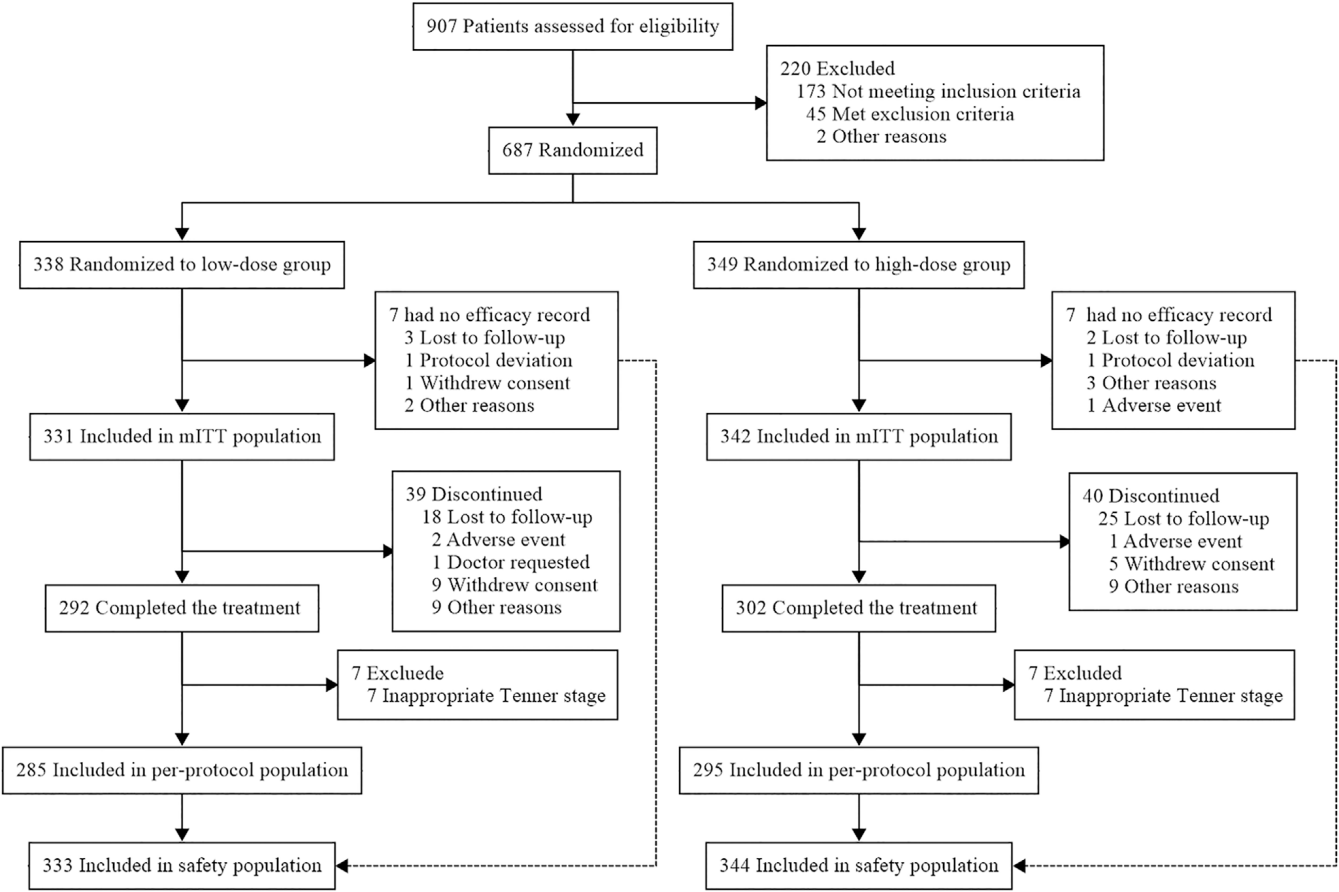
Patient flow diagram.

Between October 2014 and December 2017, 907 patients were screened and 687 patients were randomly assigned to receive 0.14 mg/kg/week of PEG-rhGH (*n* = 338) or 0.2 mg/kg/week (*n* = 349) (Figure 1). Seven patients in each group had no efficacy records and were not included in the mITT population. A total of 594 patients completed all follow-up visits, and seven patients in each group were excluded during data verification due to puberty. Finally, 285 patients in the low-dose group and 295 in the high-dose group were included in the per-protocol population. Since the results of the efficacy analysis of the per-protocol population were similar to those of the mITT population, only the mITT results are presented.

The demographic and baseline characteristics of the study population were comparable between the treatment groups (Table 1). All the patients were preadolescents, and BA/CA indicated retardation of bone maturation. All subjects were negative for Anti-GH antibodies.

**TABLE 1:** Demographic and clinical characteristics at baseline (mITT).

Characteristics	Low-dose group (*N* = 331)	High-dose group (*N* = 342)	*p* value
Male/Female	235/96	224/118	0.1256
CA, year	7.48 ± 2.48	7.48 ± 2.47	0.9739
BA, year	5.31 ± 2.27	5.36 ± 2.23	0.7979
BA/CA	0.69 ± 0.13	0.70 ± 0.12	0.7575
Height, cm	111.26 ± 12.30	111.51 ± 12.64	0.7929
Weight, kg	19.71 ± 5.50	19.62 ± 5.54	0.8367
BMI, kg/m^2^	15.65 ± 1.85	15.48 ± 1.75	0.2273
Peak GH, ng/mL	5.92 ± 2.63	5.99 ± 2.54	0.7270
HV, cm/year	2.31 ± 1.54	2.44 ± 1.48	0.2746
Ht SDS	−2.68 ± 0.85	−2.66 ± 0.72	0.7271
IGF-1, ng/mL	120.43 ± 70.82	113.32 ± 60.93	0.2193
IGF-1 SDS	−1.15 ± 1.48	−1.10 ± 1.46	0.6398

CA, chronological age; BA, bone age; BMI, body mass index; Ht, height; HV, height velocity; IGF-1, insulin-like growth factor-1.

### Efficacy assessment

**FIGURE 2:**
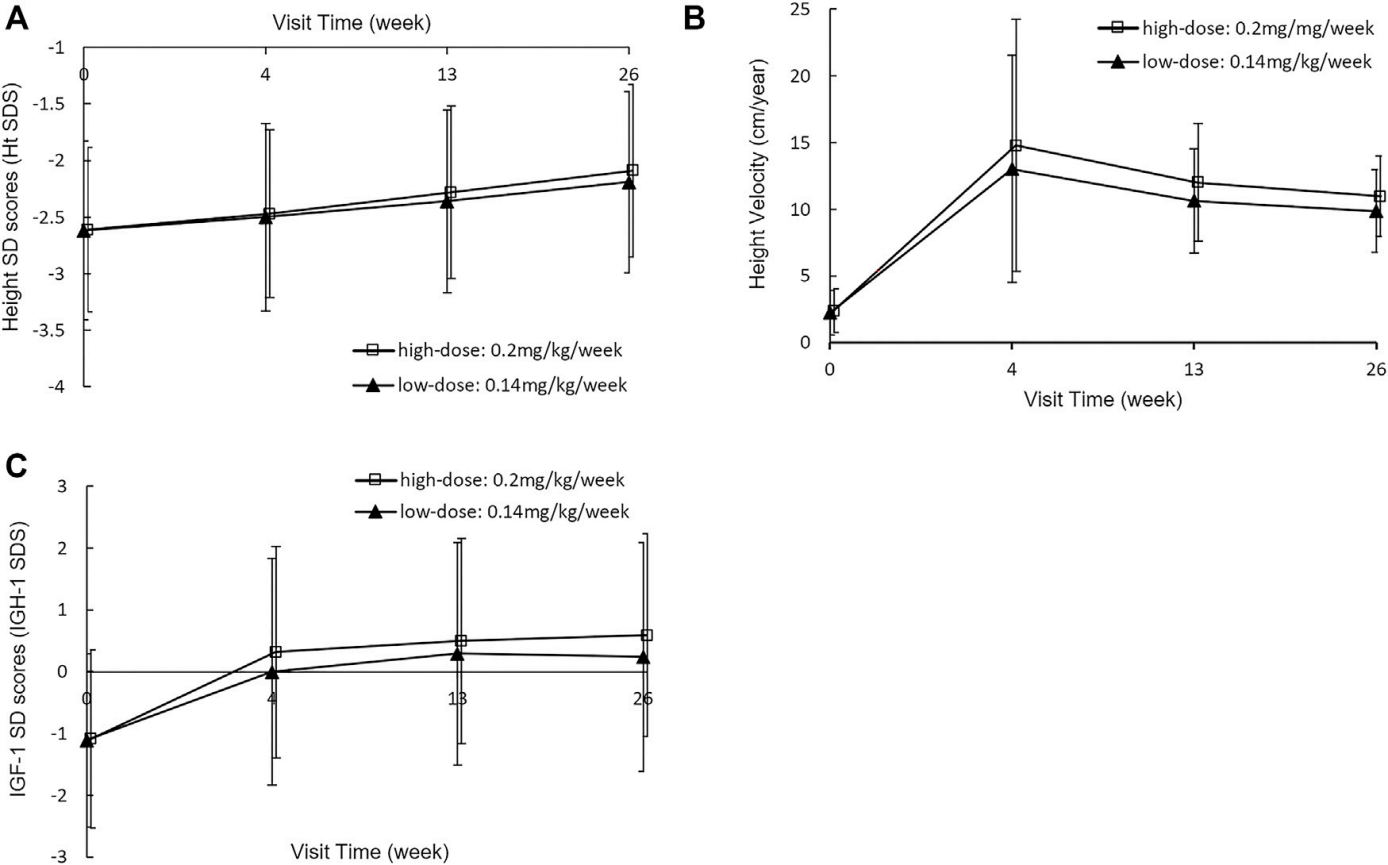
Height SDS **(A)**, Height velocity **(B)** and IGF-1 SDS **(C)** at baseline and week 4, 13 and 26 with a PEG-rhGH dose of 0.14 mg/kg/week or 0.2 mg/kg/week.

After PEG-rhGH treatment, the mean Ht SDS increased significantly in both dose groups at each assessment (Figure 2A). It increased from −2.68 ± 0.85 at baseline to −2.25 ± 0.72 at week 26 (*p* < 0.0001) in the low-dose group and from −2.66 ± 0.72 to −2.14 ± 0.75 (*p* < 0.0001) in the high-dose group. At each visit, the mean increments of Ht SDS in the low-dose group and the high-dose group were 0.12 ± 0.12 vs*.* 0.14 ± 0.13 (*p* = 0.1302) at week 4, 0.27 ± 0.19 vs*.* 0.32 ± 0.18 (*p* = 0.0008) at week 13, and 0.42 ± 0.28 vs*.* 0.51 ± 0.25 (*p* < 0.0001) at week 26, respectively. This suggests that the improvement of Ht SDS is dose-dependent, and the high dose had a more significant improvement in linear growth than the low dose. The lower limit of 95% CI of the Ht SDS change difference was −0.13, which was below the margin of −0.10. Thus, non-inferiority of Ht SDS change was not established (*p* = 0.2987).

HV increased rapidly in the first 4 weeks of PEG-rhGH treatment in both groups and then decreased slowly (Figure 2B). Similar effects of PEG-rhGH were observed in the HV as was observed in the Ht SDS. HV increased at a rate of 10.89 ± 8.05 cm/year in the low-dose group and 11.91 ± 8.83 cm/year in the high-dose group at week 4 (*p* = 0.1163). Then the increments decreased to 8.58 ± 4.30 cm/year in the low-dose group and 9.39 ± 8.97 cm/year in the high-dose group at week 13 (*p* = 0.0108), and 7.73 ± 3.53 cm/year and 8.46 ± 2.99 cm/year at week 26 (*p* = 0.0042). The low-dose group met the non-inferiority compared with the high-dose group, with a lower limit of 95% CI of −1.22 within the non-inferiority margin (*p* < 0.0001).

The mean IGF-1 SDS values also increased significantly (Figure 2C). In the low-dose group, it increased from -1.15 ± 1.48 at baseline to 0.06 ± 1.89, 0.20 ± 1.78, and 0.19 ± 1.82 at week 4, 13, and 26, respectively. And in the high-dose group, it increased from -1.10 ± 1.46 at baseline to 0.26 ± 1.74, 0.45 ± 1.68, and 0.57 ± 1.68 at week 4, 13, and 26, respectively. There were no significant increases of IGF-1 SDS at week 13 and 26 from baseline between two groups (*p* = 0.9508 in the low-dose group and *p* = 0.3766 in the high-dose group).

### Safety

A total of 677 patients were concluded for the Safety analysis: 333 in the low-dose group and 344 in the high-dose group. Anti-GH antibodies were tested for patients at week 13 and week 26 after treatments initiation. No positive anti-drug antibodies were detected in both two groups. There were no statistical differences in the incidence of AEs and SAEs between the two groups (AEs: 50.5% vs*.* 53.9%, *p* = 0.3963; SAEs: 1.2% vs*.* 1.7%, *p* = 0.7525). The most common AEs were upper respiratory tract infections, followed by cough and fever in both the low-dose group (31.5%, 8.7%, and 7.8%) and the high-dose group (33.5%, 8.9%, and 6.5%). 33 in the low-dose group and 31 in the high-dose group were considered to be PEG-rhGH-related (*p* = 0.6896). All SAEs were not PEG-rhGH-related except for one case of Henoch-Schonlein purpura in the low-dose group, where the correlation with PEG-rhGH was not identifiable.

During PEG-rhGH treatment, no statistical changes were found in blood glucose and lipid indexes including fasting blood glucose, fasting insulin, glycosylated hemoglobin, total cholesterol, triglycerides, high-density lipoprotein, low-density lipoprotein (*p* > 0.05) (Table 2).

**TABLE 2:** Blood glucose and lipid indexes during PEG-rhGH treatment (SS).

Index	Visit point, week	Low-dose group (*N* = 333)	High-dose group (*N* = 344)
FPG, mmol/L	0	4.73 ± 0.54	4.80 ± 0.60
	26	4.76 ± 0.52	4.82 ± 0.46
INS1, mIU/L	0	5.71 ± 4.87	5.97 ± 4.95
	26	6.28 ± 5.36	6.28 ± 4.66
HbA1c, %	0	5.35 ± 0.37	5.30 ± 0.87
	26	5.37 ± 0.35	5.32 ± 0.39
TC, mmol/L	0	4.34 ± 0.81	4.32 ± 0.81
	26	4.45 ± 0.89	4.38 ± 0.90
TG, mmol/L	0	0.79 ± 0.35	0.81 ± 0.44
	26	0.80 ± 0.37	0.80 ± 0.36
HDL, mmol/L	0	1.55 ± 0.33	1.54 ± 0.32
	26	1.55 ± 0.33	1.54 ± 0.38
LDL, mmol/L	0	2.38 ± 0.71	2.35 ± 0.67
	26	2.41 ± 0.70	2.37 ± 0.72

FPG, fasting blood glucose; HbA1c, glycosylated hemoglobin; INS1, fasting insulin; TC, total cholesterol; TG, triglyceride; HDL, high-density lipoprotein; LDL, low-density lipoprotein.

## Discussion

Jintrolong^®^ is the first long-acting rhGH preparation approved by the Center for Drug Evaluation of China. Based on the results of the present study, PEG-rhGH is effective and safe at a lower dose of 0.14 mg/kg/week for improving Ht SDS, HV and IGF-1 SDS in children with GHD; non-inferiority of Ht SDS at the dose of 0.14 mg/kg/week was not established after 26 weeks of treatment.

The GH/IGF-1 axis is critical for growth regulation (Balhara et al., 2012). GH induces bone growth by stimulating the production of IGF-1 in the liver, which in turn regulates GH secretion and stimulates longitudinal bone growth in the growth plate (Balhara et al., 2012; Wu et al., 2015). After 26 weeks of PEG-rhGH treatment, significant increases in Ht SDS and HV were observed in both dose groups, as expected. The incremental changes in Ht SDS and HV in the high-dose group at week 26 yielded similar results as were reported in a Phase IV clinical trial and another single-center, nonrandomized cohort study of Jintrolong^®^ at the same dose of 0.2 mg/kg/week (Qiao et al., 2019; Sun et al., 2021), but were less than the results from the Phase III clinical trial of Jintrolong^®^ (Luo et al., 2017). Changes in Ht SDS were negatively correlate with age, baseline IGF-1, and peak GH levels (Sun et al., 2021). The mean peak GH level and mean value of IGF-1 SDS were lower in the Phase III clinical trial, which may explain the differences in growth responses in different clinical trials. Attempts have been made to extend the dosing interval of PEG-rhGH, however, changes of both Ht SDS and HV failed the non-inferiority test to weekly administration of PEG-rhGH or daily administration of rhGH (Sun et al., 2021). There were also some clinical trials that use the HV improvement as the primary efficacy outcome with the non-inferiority margin of −2 cm (Luo et al., 2017; Czepielewski et al., 2019). Although the non-inferiority was not established in terms of improving Ht SDS change, our results demonstrated that the dose of 0.14 mg/kg/week was non-inferior to the dose of 0.2 mg/kg/week in improving HV of children with GHD. Meanwhile, the efficacy of PEG-rhGH treatment at 0.14 mg/kg/week were consistent with the conventional dose of rhGH treatment in previous studies (Xue et al., 2016; Deal et al., 2018; Czepielewski et al., 2019). These results suggest that the PEG-rhGH dose of 0.14 mg/kg/week could be considered as a low dose option to attain an optimistic efficacy, which would reduce both adverse reactions and the treatment costs.

The serum IGF-1 level is an important parameter for monitoring GH treatment. It has been reported that children whose rhGH doses were adjusted maintain serum IGF-1 levels in the upper normal range (+ 1.5- + 2.5 SD) gained better improvement in growth response compared to children with IGF-I levels in the mid-normal range (Cecconi et al., 2004; Cohen et al., 2007; Pfäffle, 2015). Similar to Ht SDS and HV, IGF-1 SDS was significantly elevated during the 26 weeks and showed dose-dependent changes. Notably, the IGF-1 SDS rapidly increased at week four and reached a plateau at week 13 in the low-dose group but continued to increase slightly in the next 5 months in the high-dose group, although no significant differences observed (*p* > 0.05). The trends were different from the Phase III clinical trial of Jintrolong^®^, in which IGF-1 SDS reached a plateau at around week 13 and then gradually decreased with the same dose of 0.2 mg/kg/week (Luo et al., 2017). This difference may be attributed to the huge inter-individual variation in IGF-1 levels which are influenced by sex, age, body weight, nutritional status, and puberty stage and so on (Liu et al., 2019; Witkowska-Sędek et al., 2019; Papathanasiou et al., 2021). The previously mentioned cohort study reported that IGF-1 SDS in the PEG-rhGH group reached the upper limit of the normal range (0.96 ± 1.39) during the first 6 months and continued to increase over the next 18 months (Qiao et al., 2019). Considering the risks associated with IGF-1, the question of whether a high dose of PEG-rhGH leads to a supraphysiological level of IGF-1 requires long-term follow-up.

GH activates insulin-sensitive lipase, promotes fat decomposition, inhibits glucose uptake and utilization in skeletal muscles and adipose tissue, reduces glucose consumption, and increases blood glucose levels (Weber et al., 2017). It has been observed that the blood glucose and lipid levels decrease after rhGH treatment (Ciresi et al., 2007; Slattery et al., 2014; Kubo et al., 2017), while other clinical trials have not found significant changes in glucose metabolism after rhGH treatment (Czepielewski et al., 2019). A recent meta-analysis revealed a favorable role of rhGH therapy in lipid metabolism, which might depend on the duration of the intervention; however, the role of rhGH in glucose metabolism was not significant (Yuan et al., 2021). For instance, an increase in HbA1c level was observed after 1 year of rhGH therapy in a retrospective study of 101 pediatric patients with GHD (Pellegrin et al., 2019). For PEG-rhGH, improvements in lipid profiles (Hou et al., 2016) and non-significant changes in lipid metabolism (Wang et al., 2021) have been reported; however, none of them exert an unfavorable effect on glucose metabolism (Hou et al., 2016; Qiao et al., 2019; Wang et al., 2021). In our study, no significant changes were found in glucose or lipid metabolism after 26 weeks of PEG-rhGH treatment in children with GHD, regardless of PEG-rhGH dose. Our metabolomics analysis had revealed a strong association between fatty acids metabolism and the clinical efficacy of PEG-rhGH therapy, which would likely to be involved in fatty acid metabolism and energy metabolism (Li et al., 2022). Long-term follow-up is needed to confirm the effects of PEG-rhGH treatment on glucose and lipid metabolism.

Although the short-term efficacy and safety of PEG-rhGH treatment has been proven in clinical trials, introduction of a modified PEG molecule may cause new side effects (Lal and Hoffman, 2018). In addition, long-term elevated GH levels produced by PEG-rhGH treatment may induce iatrogenic acromegaly, neoplasia and glucose intolerance (Yuen et al., 2021). Therefore, every centimeter gained from PEG-rhGH treatment comes with a certain amount of risk. Moreover, despite the reduced frequency of injections, the cumulative cost of long-term treatment with PEG-rhGH remains high. Further pharmacoeconomic evaluations are needed to determine the correct cost and risk-benefit ratio.

Our study has some limitations. First, the pharmacokinetic and pharmacodynamic profiles of 0.14 mg/kg/week dosing have not been evaluated in order to explain the differences in the IGF-1 responses between the two PEG-rhGH doses. Second, serum IGF-1 levels increased steadily after PEG-rhGH injection and reached to a peak concentration after 2 days (Hou et al., 2016). However, for the convenience of patients and their parents, the follow-up visit was not strictly set for the second day after dosing according to the study protocol, which might introduce some error in the accuracy of the IGF-1 SDS.

In conclusion, there were significant increases in Ht SDS, HV, and IGF-1 SDS at week 26 after PEG-rhGH treatment in both dose groups. Ht SDS improvement with treatment using 0.14 mg/kg/week of PEG-rhGH was not non-inferiority to that at the standard dose of 0.2 mg/kg/week. Additionally, there were no significant changes in glucose or lipid metabolism after PEG-rhGH treatment at the different doses. Furthermore, a longer follow-up period is needed to assess the long-term efficacy and safety of lower doses of PEG-rhGH to optimize the therapeutic dose of PEG-rhGH.

## Data Availability

The original contributions presented in the study are included in the article/Supplementary Material, further inquiries can be directed to the corresponding authors.

## Abbreviations

AE, adverse event; BA, bone age; CA, chronological age; GH, growth hormone; GHD, growth hormone deficiency; Ht, height; HV, height velocity; IGF-1, insulin-like growth factor-1; mITT, modified intention-to-treat; PEG-rhGH, polyethylene glycol-recombinant human growth hormone; rhGH, recombinant human growth hormone; SD, standard deviation; SDS, standard deviation score; SS, safety set.
